# High mortality at safety-net hospitals for patients with cirrhosis

**DOI:** 10.1097/HC9.0000000000000964

**Published:** 2026-06-12

**Authors:** Sachin Shah, Mignote Yilma, Sabrina Omer, Amy Shui, Nicole J. Kim, Kali Zhou, Michele Tana, Neil Mehta

**Affiliations:** 1 Department of Medicine, University of California San Francisco, San Francisco, California, USA; 2 Department of General Surgery, University of California San Francisco, San Francisco, California, USA; 3 Division of Gastroenterology, University of Washington, Seattle, Washington, USA; 4 Department of Epidemiology and Biostatistics, University of California San Francisco, San Francisco, California, USA; 5 Division of Gastrointestinal and Liver Disease, University of Southern California, Los Angeles, California, USA; 6 Department of Gastroenterology and Hepatology, Zuckerberg San Francisco General Hospital, San Francisco, California, USA; 7 Department of Gastroenterology, University of California San Francisco, San Francisco, California, USA

Dear Editors,

Safety-Net Hospitals (SNHs) provide a significant amount of healthcare and related services to patients without insurance or who are otherwise medically and socially vulnerable. Our multicenter SNH study provides complementary data to the article recently published by Kim et al, titled “Feasible and Acceptable Social Drivers of Health Screening Among Patients With Chronic Liver Disease” by answering why screening is needed based on longitudinal mortality data from high-risk populations across multiple geographic locations, identifying a large gap in liver transplant (LT) referral, and highlighting documentation status as an additional social determinant of health (SDOH) to consider for screening.^1^

Kim et al’s^2^ study was a single-center study with 250 participants and demonstrated that screening with their locally developed screener, SINCERE, SDOH, was prevalent and had overall acceptable convergent validity in detecting social needs. We evaluated the impact of SDOH in 498 patients with cirrhosis at 3 high-volume SNHs (Los Angeles General, Harborview Medical Center, and Zuckerberg San Francisco General; Supplemental Table S1, http://links.lww.com/HC9/C362). We found that in a median follow-up time of 6.2 years, 44.6% of patients (n=222) died. Within 1 year of their initial hepatology clinic visit, we observed a 21.1% mortality, which we estimate to be higher than expected for patients with cirrhosis in non-safety-net hospitals (Supplemental Figure S1, http://links.lww.com/HC9/C362).^3^ We hope these findings emphasize the importance and need for targeted interventions such as the implementation of screening in this setting.

In addition, we document that 77.4% of deaths in this patient population occurred without LT referral. We think this importantly highlights a gap in care in the LT process. The strength of this number comes from collective data looking at referrals to multiple transplant centers and aligns with other literature suggesting a lower likelihood of LT from socioeconomically deprived neighborhoods.^4^

In addition, our multivariable analyses identified documentation status as a key SDOH not captured by the existing screener included in the SINCERE and EMR tools evaluated by Kim et al that we feel is crucial. In our study, after controlling for demographic and socioeconomic variables, unknown or missing documentation status had 1.5 times the mortality risk compared with documented patients (Table 1). Kim et al^2^ screened for food insecurity (26%), financial strain (43%), transportation needs (8%), housing instability (24%), and lack of social support (5%), which are all crucial domains in finding vulnerable patients. However, we believe that data on documentation status is important to measure, as it can affect access to LT and other specialty care. Outcomes data on patients who are unauthorized immigrants is sparse, and even more limited in the LT space. More than 11 million unauthorized immigrants reside in the United States, and overall, the national landscape points toward limited access to LT. From 2012 to 2019, the proportion of transplants allocated to unauthorized immigrants was only 0.4% (10-fold lower than the estimated unauthorized immigrant population in the United States), despite patient and graft survival among those who underwent LT being similar to that of US citizens.^5^ We believe that documentation status may be a consequential social determinant for patients with cirrhosis in a safety-net setting and an important gap that should be added to screening instruments.

**TABLE 1:** Unadjusted model and multivariable models [Model 1 (Demographic and socioeconomic factors) and Model 2 (Demographic, socioeconomic, and disease-related factors)] and risk of death in patients with cirrhosis at 3 safety-net hospitals

Variable	Hazard ratio	95% CI	*p*
Unadjusted model			
Age	1.03	1.01–1.05	*p*<0.001
Unknown/missing documentation	1.60	1.22–2.10	*p*<0.001
Hepatocellular carcinoma	2.50	1.73–3.61	*p*<0.001
Any medical comorbidity	1.81	1.23–2.68	*p*=0.003
MELD-Na	1.04	1.01–1.06	*p*=0.008
Multivariable Model 1			
Age	1.03	1.01–1.04	*p*<0.001
Unknown/missing documentation	1.50	1.14–1.97	*p*=0.004
Multivariable Model 2			
Age	1.03	1.01–1.05	*p*<0.001
Unknown/missing documentation	1.63	1.24–2.16	*p*<0.001
Hepatocellular carcinoma	2.50	1.69–3.61	*p*<0.001
MELD-Na	1.04	1.03–1.09	*p*<0.001

Abbreviation: MELD-Na, Model for End-stage Liver Disease–Sodium.

Taken together, we believe that our 2 studies form logical and complementary evidence-based data. Kim et al.^2^ show that SDOH screening in a hepatology clinic can take only 38 seconds (via SINCERE) and is acceptable to 69% of patients.^2^ Our data demonstrates that in the SNH population, where SDOH burden is highest, mortality is significantly elevated, and documentation status, which is not currently being screened for, is associated with mortality. We hope that with our study, future SDOH screening instruments incorporate documentation status and early liver transplant protocols for safety-net populations.

In conclusion, our multicenter data on mortality in SNH demonstrates that documentation status accounts for access to LT referral and is an important SDOH domain for measurement to understand predictors of mortality in cirrhosis. We believe this research will not only demonstrate that SDOH screening is feasible, but also the need to expand tools and address the high mortality in our most vulnerable patients.

## Supplementary Material
