# Supplementary material for: High mortality at safety-net hospitals for patients with cirrhosis
Source: Hepatol Commun. 2026 Jun 12;10(7):e0964. doi: 10.1097/HC9.0000000000000964 (PMC13263105; doi:10.1097/HC9.0000000000000964)
Supplement: Supplementary file 1 [file hc9-10-e0964-s001.docx]

**Supplemental Table 1**. Patient Characteristics at Three Safety-Net Hospitals

| **Variable** | **Overall n (%)** |
| --- | --- |
| **Total Patients** | 498 (100%) |
| **Patient Variables** | |
| Mean Age (±SD) | 53.2 (9.8) |
| Sex | |
| Men | 351 (70.5%) |
| Women | 147 (29.5%) |
| Race and Ethnicity | |
| Hispanic of Latinx | 299 (60.0%) |
| Non-Hispanic Black | 31 (6.2%) |
| Non-Hispanic White | 105 (21.1%) |
| Non-Hispanic Other | 37 (7.4%) |
| Unknown/Missing | 26 (5.2%) |
| Marital Status | |
| Divorced, separated, or widowed | 101 (20.3%) |
| Married | 148 (29.7%) |
| Single | 234 (47.0%) |
| Unknown/Missing | 15 (3.0%) |
| Documentation Status | |
| Documented | 270 (54.2%) |
| Undocumented | 28 (5.6%) |
| Unknown/Missing | 200 (40.2%) |
| Foreign Born Status | |
| No | 62 (12.4%) |
| Yes | 226 (45.4%) |
| Unknown/Missing | 210 (42.2%0 |
| **Socioeconomic Status Variables** | |
| Housing | |
| Stable | 327 (65.7%) |
| Unstable | 64 (12.9%) |
| Unknown/Missing | 107 (21.5%) |
| Insurance Type | |
| Medicaid | 318 (63.9%) |
| Medicare | 81 (16.3%) |
| Private | 9 (1.8%) |
| Uninsured | 71 (14.3%) |
| Unknown/Missing | 19 (3.8%) |
| **Liver Disease Variables** | |
| Etiology | |
| Alcoholic Liver Disease | 252 (51.0%) |
| Metabolic Dysfunction-Associated  Steatotic Liver Disease | 28 (5.7%) |
| Hepatitis C Virus | 81 (16.4%) |
| Hepatitis B Virus | 14 (2.8%) |
| Other | 31 (6.3%) |
| Decompensation | |
| Ascites | 363 (72.9%) |
| Hepatic Encephalopathy | 181 (36.3%) |
| Variceal Bleed | 78 (15.7%) |
| Diagnosis of Hepatocellular Carcinoma | 47 (9.5%) |
| Mean Meld at Baseline (± SD) | 20.1 (4.9) |

1.
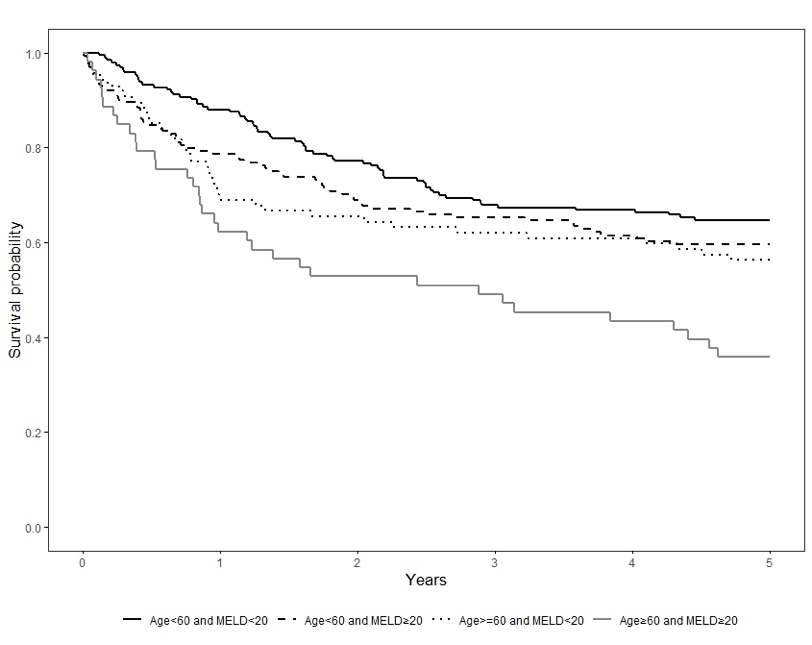


Product-Limit Survival Estimates with Number of Subjects at Risk

Product-Limit Survival Estimates with Number of Subjects at Risk

1.
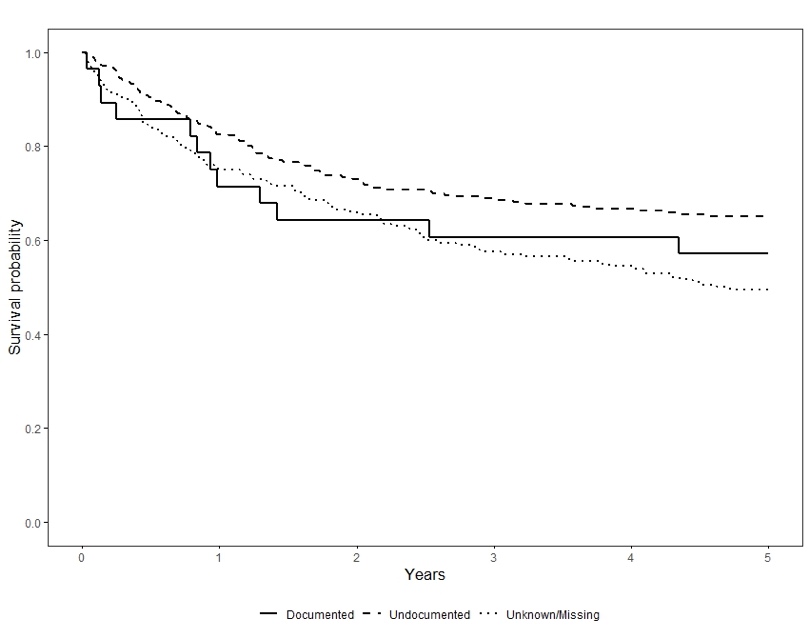


**Supplemental Figure 1**. (A) Kaplan Meier Survival Curves Following Initial Outpatient Hepatology Visit in Patients with Cirrhosis at Three Safety Net Hospitals Stratified by Age and MELD-Na (B) and Stratified by Documentation Status.
